# Bacterial swimming in porous gels exhibits intermittent run motility with active turns and mechanical trapping

**DOI:** 10.1038/s41598-025-02741-1

**Published:** 2025-06-27

**Authors:** Agniva Datta, Sönke Beier, Veronika Pfeifer, Robert Großmann, Carsten Beta

**Affiliations:** 1 University of Potsdam, Institute of Physics and Astronomy, Potsdam, 14476 Germany; 2 Kanazawa University, Nano Life Science Institute (WPI-NanoLSI), Kanazawa, 920-1192 Japan

**Keywords:** Cellular motility, Biological physics, Bacteria, Statistical physics

## Abstract

While bacterial motility has been well characterized in uniform liquids, only little is known about how bacteria propagate through complex environments, such as gel-like materials or porous media that are typically encountered in tissue or soil. Here, we study bacterial swimming in polysaccharide matrices formed by different concentrations of agar. We focus on the soil bacterium *Pseudomonas putida* (*P. putida*) that is known for its multimode swimming pattern, where a polar bundle of flagella may push, pull, or wrap around the cell body. In the gel matrix, *P. putida* cells display run-and-turn motility with exponentially distributed run times and intermittent turning phases that follow a dwell time distribution with power-law decay. An analysis of the turn angle distribution suggests that both, flagella mediated turning as well as mechanical trapping in the agar matrix are part of the overall swimming pattern. We compare these results to knockout mutants which differ from the wild-type in their swimming speed and show altered probabilities for the occurrence of the three swimming modes. Their run length distributions in the agar matrix are, however, identical demonstrating that run episodes of bacterial swimmers in a gel matrix are primarily determined by the surrounding geometry. We propose a minimal active particle model providing analytical solutions that quantitatively explain the observed time dependence of the mean squared displacement in the gel based on the experimentally observed motility pattern and the measured waiting-time distributions.

## Introduction

Bacterial swimming is one of the most common forms of motility at the cellular level^1^. For many decades, swimming in uniform liquid environments has been studied, mostly focusing on the classical example of *E. coli* that moves in a sequence of straight runs, interrupted by motor-induced abrupt tumbling events^2^. However, in many natural habitats, such as mucus, soil, or plant and animal tissues, bacterial swimmers face heterogeneities and strong confinement. Compared to free swimming in a uniform liquid, much less is known about how individual cells navigate such complex environments.

To address this question, bacterial swimmers were studied in well-defined microfluidic geometries and other artificial compartments^3–5^, taking also the impact of fluid flow into account^6^. Also, disordered porous media were considered, in which *E. coli* cells move in directed hops that are interrupted by transient mechanical trapping events in narrow spaces or cavities of the porous material^7,8^. However, in many habitats, such as the human gut or the plant rhizosphere, bacteria move through layers of mucus and other gelatinous matrices. While some studies have explored these aspects^9–11^, a comprehensive quantitative analysis that aligns experimental observations with mathematical models to decipher bacterial swimming in gel-like surroundings and its impact on overall spreading is still missing. This is, however, a key prerequisite to understand how pathogens invade host cells and tissue^12–15^ or how rhizobia colonize plant roots in the soil^16–18^.

Studies of bacterial swimming in bulk liquid have shown that, besides the classical run-and-tumble paradigm of *E. coli*, a variety of different swimming modes and turning maneuvers can be observed in other species. During runs of peritrichously flagellated *E. coli*, cells always move in push mode, with the flagellar bundle propelling the cell body from behind. In contrast, polarly flagellated species may also switch to pull mode or perform screw thread motility with their flagellar helix wrapped around the cell body^19^. Sharp directional reversals, flagellar flicking and wrapping are turning maneuvers observed along with these different swimming modes^20^. The motility pattern of these bacteria determines their dispersal as well as their chemotaxis^19,21,22^. But how it influences motility in complex environments is still not fully understood.

Here, we study the soil bacterium *Pseudomonas putida* (*P. putida*) based on single cell trajectory recordings in a gel-like polysaccharide matrix formed by semisolid agar. The macroscopic rheological properties of agar and the related agarose, such as storage and loss moduli, have recently been characterized experimentally^23,24^. The gel network forms a porous medium in which the average pore size is in the range of 740 nm to 4800 nm for $0.25 \, \%$ agar^10^. Thus, the pores are wide enough for the bacteria, which are approximately 2 $\upmu$m long and 1 $\upmu$m wide^25^, to swim through. However, due to the variable and disordered geometry, bacteria may intermittently get trapped in the dense agar matrix. *P. putida* propels itself with a tuft of flagella polarly attached to one end of the rod-shaped cell body^25^. It can swim in push, pull, or wrapped mode, interrupted by directional reversals or stop events^26,27^. The wrapped mode was observed to play a key role in chemotaxis^22^, it is more likely to occur under conditions of increased viscosity and mechanical confinement, and its occurrence can be tuned by introducing knockout mutations in the torque generating MotAB and MotCD stator units of the flagellar motor^28^.

We recorded the motility of *P. putida* KT2440 wild-type cells, as well as $\Delta$*motAB* and $\Delta$*motCD* mutant cells^28^ in two different concentrations of semisolid agar with phase-contrast microscopy at a frame rate of 20 frames per second (see Supplementary Movies). Recordings of a total duration of 60 s were segmented to identify the positions of cells in each time frame. Cell trajectories were then extracted from the time lapse image sequences by linking cell positions in successive frames using a next neighbor particle tracking algorithm^29^. In addition, we performed high-resolution dual color fluorescence microscopy at a rate of 100 frames per second to identify the different swimming modes. Here, the flagella were labeled with Alexa 488 C$_5$ maleimide and the cell body was stained with a red membrane intercalating dye. For details of cell culture, microscopy recordings, and image analysis, see Methods.

## Results

**Fig. 1:**
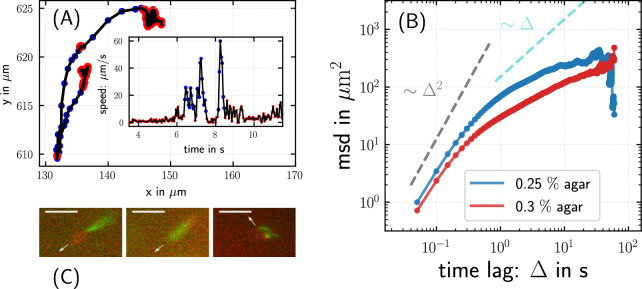
Trajectories reveal intermittent run-motility of bacteria in agar gel. (**A**) Trajectory of a bacterium in $0.25 \,\%$ agar, with the corresponding speed as a function of time as an inset. Runs are indicated in blue. (**B**) MSD of *P. putida* wild-type in $0.25\,\%$ and $0.3\,\%$ agar, showing a crossover from ballistic to subdiffusive scaling. (**C**) The three swimming modes push, pull, and wrapped in semisolid agar are shown for fluorescently labeled cells. The scale-bar corresponds to 5 $\upmu$m.

### Bacterial swimmers display intermittent run motility in agar gels

Visual inspection of trajectories revealed that they are typically composed of straight runs, interrupted by phases during which the bacterium hovers around a fixed position before performing the next run in a different direction, see Fig. 1A for an example trajectory. Given the extended duration of many of these episodes in comparison to the short turn maneuvers (stops and reversals) observed in open liquid^22,26^, we assume that they are associated with mechanical trapping in the gel matrix, during which cells are essentially immobile until they free themselves again, often via a reversal. In order to separate runs from immobile episodes (hereafter referred to as events), we applied k-means clustering^30^ on the speed of the bacterium and the change of direction of motion (see Supplementary Note 1 for details). Our fluorescence microscopy recordings demonstrated furthermore that all three swimming modes—push, pull, and wrapped—that are known from motility in bulk liquid are also present in the porous environment, as shown in Fig. 1C. This is relevant as transitions between the swimming modes are associated with changes in the arrangement of the flagellar bundle which, in turn, lead to characteristic turning maneuvers such as stops and reversals^22,27^. As all swimming modes are observed in semisolid agar, the flagella-mediated turning maneuvers known from bulk liquid are expected to be present in semisolid agar as well.

### Mean squared displacement shows crossover from ballistic to sublinear scaling

The mean squared displacement (MSD), i.e. the mean squared distance $m_2$ traversed by a bacterium in a time $\Delta$, is displayed in Fig. 1B for *P. putida* wild-type cells in $0.25\,\%$ and $0.3\,\%$ agar. For both agar concentrations, the time evolution of the MSD shows a similar characteristic, namely a crossover around $\Delta \approx 0.5 \, \mbox{s}$ from ballistic motion with $m_2 \sim \Delta ^{\!\!\,2}$ at short times to a sublinear regime at longer times; at those intermediate timescales, the MSD scales subdiffusively. Subdiffusive scaling of the MSD is not observed for bacterial motility in bulk liquid and is, hence, inherently related to the disordered nature of the agar gel.

### Run times are exponentially distributed while dwell times show a power law decay

**Fig. 2:**
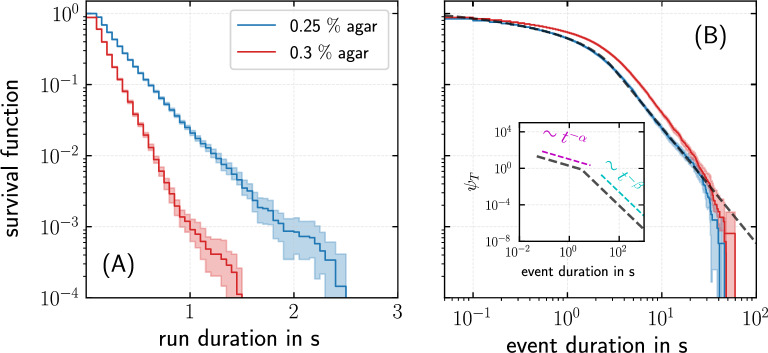
Run times are exponentially distributed, whereas dwell times follow a power-law distribution. The two panels show the survival functions $\int _{t}^{\infty } dt' \, \psi _{R,T}(t')$ of the sojourn times of (**A**) runs and (**B**) immobile episodes (the dotted line corresponds to a fit of the survival function of a piecewise power-law distribution); the inset in (**B**) illustrates the piecewise power-law with the scaling of $\psi _T(t)$ with $\psi _T \propto t^{-\alpha }$ at intermediate timescales ($\alpha = 0.79$) and $\psi _T \propto t^{-\beta }$ with $\beta =2.63$ for large times. Exponents were inferred by a least-square fit to the survival function. We obtained the mean run times $\langle t_R \rangle = 0.35\, \mbox{s}$ and $0.23\,\mbox{s}$ and mean dwell times $\langle t_T\rangle = 2.07\,\mbox{s}$ and $3.63\,\mbox{s}$ for *P. putida* wild-type cells in $0.25\, \%$ and $0.3\, \%$ agar, respectively. The shaded regions correspond to the error bars ($95\, \%$ confidence interval) associated with the survival functions obtained by bootstrapping^31^.

Based on the distinction between runs and immobile episodes, we also determined the distribution of sojourn times in each state by non-parametric maximum-likelihood estimation^32^. In the following, we denote these waiting-time distributions $\psi _R(t)$ and $\psi _T(t)$, respectively. In Fig. 2A, the distribution of run times $\psi _R(t)$ is displayed in the form of a survival function. The semi-logarithmic plot reveals an exponential scaling. The mean run time is one order of magnitude smaller than the corresponding run times in homogeneous liquids–reflecting the mean free path in the surrounding gel environment, which restricts the ability of bacteria to move actively. In contrast, the distribution of dwell times $\psi _T(t)$ in immobile phases is well approximated by a piecewise power-law with an exponent $\alpha < 1$ at intermediate timescales, followed by a steep cutoff towards larger dwell times with an exponent of $\beta > 2$, see Fig. 2B, Supplementary Note 1 and Supplementary Fig. S3.

Comparing the two agar concentrations of $0.25 \, \%$ and $0.3 \, \%$, we find that the mean run time decreases significantly as the agar concentration is increased. In contrast, the distribution of dwell times during immobile episodes is almost independent of the agar concentration. Therefore, we conclude that the nature of traps does not differ between the two agar concentrations but the rate of occurrence of trap events increases for increasing agar concentration due to a decrease in pore size. Experimental recordings suggest that bacteria often leave traps in the same direction in which they entered a trap. This leads us to hypothesize that bacteria typically escape from traps by actively initiating a turning maneuver which is associated with a rearrangement of the flagellar bundle, independent of the agar concentration (cf. paragraph below and Discussion section).

### Swimming is affected by both flagella mediated turning and transient trapping in the agar

**Fig. 3:**
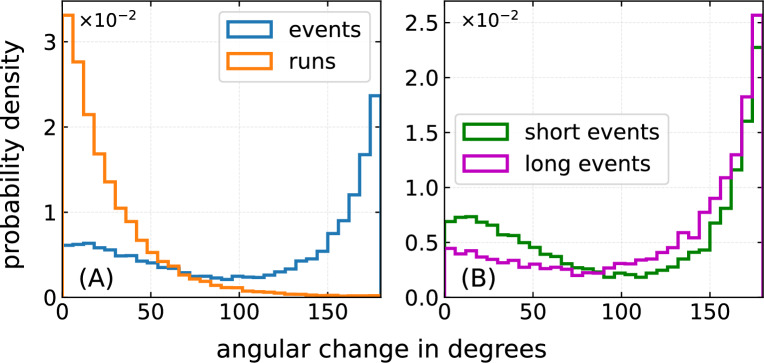
Angular change of *P. putida* in agar during runs and events. (**A**) Change in the direction of motion during runs ($\text {orange}$) and events (blue). (**B**) Turn angle distributions of short (duration shorter than $1 \, \text {s}$, shown in green) and long events (duration longer than $1 \, \text {s}$, shown in purple) for *P. putida* wild-type in $0.25 \, \%$ agar. Similar distribution functions are observed for the mutant strains and in $0.3 \, \%$ agar (cf. Supplementary Figure S2 and S5). Qualitatively, the results do not depend on the chosen cutoff value for the classification into short and long events; corresponding histograms for different cutoffs are shown in Supplementary Note 3, Fig. S8.

In bulk liquid, the motility pattern of *P. putida* is characterized by highly persistent runs that are interrupted by immobile phases of different type: bacteria may stop, without a considerable change in their direction of motion, or may reverse their swimming direction^26^. Stops and reversals are induced by changes in motor activity and are accompanied by rearrangements of the flagellar bundle, resulting in transitions between the different swimming modes^22,27^ (push, pull and wrap, illustrated in Fig. 1C). To address the influence of semisolid agar on flagella-driven motility, we measured the change in propagation direction during runs and immobile phases. We found that runs, as expected, exhibit a high directional persistence, see the corresponding curve in Fig. 3A which illustrates the difference between the direction of motion at the beginning and the end of a run. The distribution of angles reveals that the angular change during a run remains small and peaks around $0^{\circ }$. Furthermore, we characterized the fluctuations in the direction of motion between consecutive frames, which are well described by a narrow Gaussian (see Supplementary Note 1, Fig. S4).

Large directional changes are, on the other hand, expected when trapping is caused by dead ends and cavities. In these cases, cells often have to leave a trap via the same way through which they entered. We subsume the entirety of all immobile phases, including stops, reversals and mechanical traps as “events”. The change in the direction of motion during an event, defined as the difference in the orientation of two subsequent runs that were interrupted by the event, shows a broad distribution with a pronounced peak at $180^{\circ }$ (see Fig. 3A).

To gain a better understanding of the turn angle distribution and to distinguish the influence of motor induced turn events from mechanical traps, we divided the immobile event phases into long (duration longer than 1 s) and short (shorter than 1 s) events: the histogram of the short events develops a more prominent second peak at around $0^{\circ }$, reminiscent of the bimodal turn angle histogram observed for *P. putida* when swimming freely in a uniform liquid^26^, see Fig. 3B. We thus conclude that, besides mechanical trapping in the gel matrix, also actively triggered stop and turn events may occur as part of the swimming trajectories of *P. putida* in semisolid agar. Note that the study of motor-induced turning of *P. putida* in open liquid suggests that the majority of stops and reversals are shorter than the chosen cutoff value of 1 s^22^. We checked that qualitatively the results do not depend on the chosen cutoff value; corresponding histograms for different cutoffs are shown in Supplementary Note 3.

### Stator mutants and wild-type cells show similar displacement statistics in the gel

In *P. putida*, the motor torque for rotation of the flagella is generated by two stator units, MotAB and MotCD^28,33^. An earlier study of MotAB and MotCD knockout mutants revealed that the stators directly influence the probabilities of observing the three swimming modes– push, pull, and wrapped, where the wrapped mode is assumed to form under higher motor torque. Specifically, for the $\Delta$*motAB* mutant in agar the occurrence of the wrapped mode was reduced by more than 50 % compared to the $\Delta$*motCD* mutant and the wild-type^28^. Here, we analyzed trajectories of the two knockout mutants $\Delta$*motAB* and $\Delta$*motCD* in $0.25\,\%$ agar and compared them to the analysis of the wild-type presented above.

**Fig. 4:**
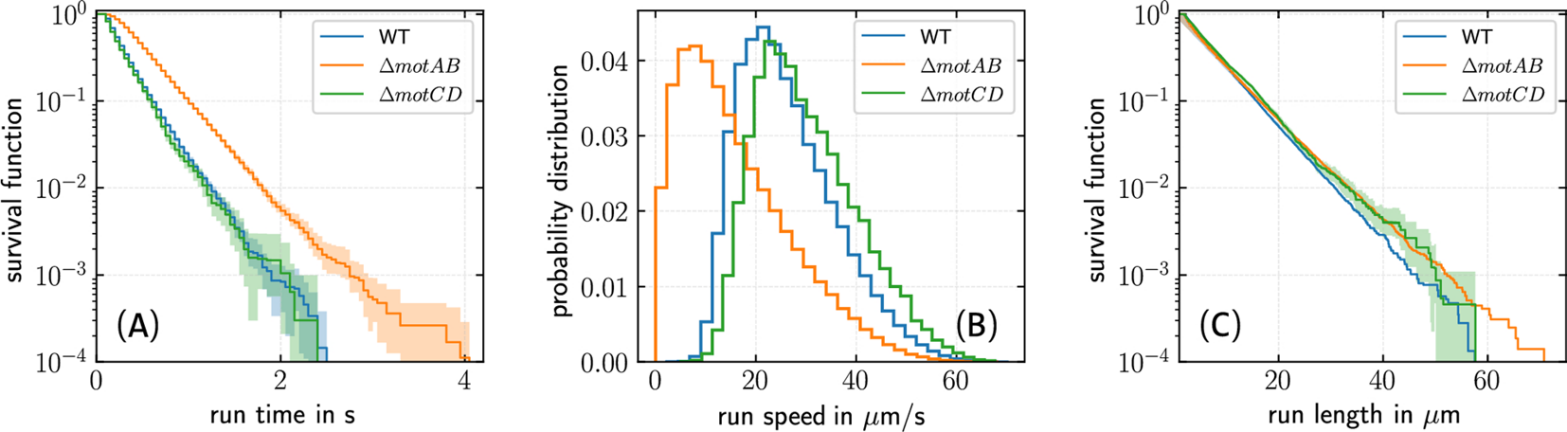
Run time, speed, and run length distributions of *P. putida* in 0.25 % agar: wild-type (WT, blue), $\Delta$*motAB* (orange), and $\Delta$*motCD* cells (green). (**A**) Survival functions of run times, (**B**) probability density functions of run speeds, and (**C**) survival functions of run lengths. The shaded regions in panels (**A**,**C**) correspond to error bars ($95\, \%$ confidence interval) obtained by bootstrapping^31^. The run length distribution decays exponentially with a mean run length of 8 $\upmu$m, comparable to the pore size^10^. In $0.3 \, \%$ agar, we found a corresponding mean run length of 5 $\upmu$m.

We observed that run times of the $\Delta$*motAB* mutant are longer on average, compared to the wild-type and the $\Delta$*motCD* mutant, see Fig. 4A. Note, however, that the average swimming speed of the $\Delta$*motAB* mutant is smaller than the speed of the wild-type and the $\Delta$*motCD* cells (see Fig. 4B), so that traveling similar distances will require longer run times for the $\Delta$*motAB* mutant. Indeed, the run length distributions are identical for all three strains (see Fig. 4C), confirming that the distances traveled in the gel are primarily determined by the geometry of the surrounding matrix and not by the details of the bacterial swimming pattern.

Further, we found that the time dependence of the MSD as well as the waiting-time distributions closely match the results observed for wild-type cells, see Supplementary Information for details. As the three swimming modes occur with different probabilities in wild-type and mutant cells, the similar shape of the MSD curves (Fig. S7) indicates that bacterial displacement in the agar matrix does not depend on the specific frequencies at which the individual swimming modes are observed but is mainly determined by the structure of the surrounding matrix.

### An active particle model provides predictions for intermittent run motility

Our experimental observations suggest that the movement of *P. putida* cells in a gel-like environment resembles intermittently self-propelled particles. Based on a recently proposed modeling framework^34^, we describe the bacterial dynamics by two coupled Langevin equations:

1a $$\begin{aligned} \frac{d\vec {r}(t) }{dt}&= v(t) \! \begin{pmatrix} \cos \phi (t) \\ \sin \phi (t) \end{pmatrix} \! , \end{aligned}$$

1b $$\begin{aligned} \frac{d \phi (t)}{dt}&= \sqrt{2D_{\phi }(t)} \, \eta (t) + \zeta _{\chi }(t). \end{aligned}$$

The first equation reflects self-propelled motion at a speed *v*(*t*) along the orientation of the body axis $\phi (t)$, and the second one models orientational changes that can occur due to rotational diffusion and instantaneous jumps. The model belongs to the general class of diffusion models with time dependent parameters^34–38^. Here, the speed *v*(*t*) and the rotational diffusion coefficient $D_{\phi }(t)$ are dichotomous processes that randomly fluctuate between two values depending on the motion state (active run or immobile event). During runs, the direction of motion fluctuates, described by Gaussian white noise $\eta (t)$ in Eq. (1b). The speed $v\! \left( t\right)$ and the rotational diffusion coefficient $D_{\phi }(t)$ of the bacterium are given by non-zero values $\left( v_{0},D_{\phi }\right)$ in the run-state and by $\left( 0,0\right)$ during trap and turn phases; note that the spatial displacement during turns and traps is negligible compared to the displacement during runs. The second term $\zeta _{\chi }(t)$ in Eq. (1b) corresponds to a non-Poissonian shot noise: at the end of each turn phase, the bacterium randomly reorients with respect to its last orientation of the body axis,

2 $$\begin{aligned} \phi \rightarrow \phi + \chi , \end{aligned}$$

in which the angle $\chi$ is drawn from a turn angle distribution $p(\chi )$, cf. Fig. 3 and Supplementary Fig. S5. The duration of run and event phases are drawn from the waiting-time distributions $\psi _{R}(t)$ and $\psi _{T}(t)$, respectively. Note that the model combines runs in push, pull, and wrapped mode in one active motility state, and similarly does not distinguish stops, reversals, or mechanical trapping events but describes all immobile phases as a second single motility state. The starting time of the observation is denoted by $t_a$, the so-called aging time.

For the model outlined above, we derived an exact, analytic expression for the velocity auto-correlation function $C_{vv}\left( t_a;\Delta \right) = \left<\dot{\vec {r}}\left( t_a\right) \cdot \dot{\vec {r}}\left( t_a + \Delta \right) \right>$ in Laplace domain^34,39^:

3 $$\begin{aligned} \hat{\hat{C}}_{vv} \! \left( s;u\right)&= \frac{v_0^{2}}{\left( u+D_{\phi }\right) } \! \left[ \frac{1-\hat{\psi }_{R} \! \left( s\right) }{s \! \left[ 1-\hat{\psi }_{R} \! \left( s\right) \hat{\psi }_{T} \! \left( s\right) \right] } - \frac{\hat{\psi }_{R} \! \left( u+D_{\phi }\right) -\hat{\psi }_{R} \! \left( s\right) }{\left[ s - \left( u + D_{\phi }\right) \right] \left[ 1-\hat{\psi }_{R} \! \left( s\right) \hat{\psi }_{T} \! \left( s\right) \right] }\cdot \frac{1-\langle \cos \chi \rangle \hat{\psi }_{T} \! \left( u\right) }{1-\langle \cos \chi \rangle \hat{\psi }_{T} \! \left( u\right) \hat{\psi }_{R} \! \left( u+D_{\phi }\right) } \right] \! . \end{aligned}$$

Reorientations enter via the factor $\langle \cos {\chi } \rangle$ only; the functional form of the distribution $p(\chi )$ is irrelevant for the velocity auto-correlation function. The arguments *s* and *u* are the Laplace variables corresponding to the aging time $t_a$ and the lag time $\Delta$, respectively. From this correlation function, we can find an exact expression for the MSD $m_2\left( t_a;\Delta \right) = \langle \left| \vec {r}\!\left( t_a+\Delta \right) -\vec {r}\!\left( t_a\right) \right| ^{2}\rangle$ in Laplace domain^39^ via

4 $$\begin{aligned} \hat{\hat{m}}_{2} \!\left( s;u\right) = \frac{2}{u} \cdot \frac{\hat{\hat{C}}_{vv} \!\left( u;u\right) - \hat{\hat{C}}_{vv} \! \left( s;u\right) }{s-u} \, , \end{aligned}$$

from which $m_2\left( t_a;\Delta \right)$ can be derived by numerically performing an inverse Laplace transform^34,39^.

To explicitly compute the MSD following Eqs. (3, 4), the parameters $v_0, D_\phi$, and $\langle \cos \chi \rangle$ as well as suitable choices for the waiting-time distributions $\psi _{R,T}(t)$ are required. We fit the survival function corresponding to $\psi _{R}(t)$ as shown in Fig. 2A using a double exponential distribution. Note in this context that the statistics of run times in wrapped mode is different compared to the push and pull modes^22^ and, thus, several timescales in the run time distribution can be expected since the analysis of run times, shown in Fig. 2, does not distinguish between different swimming modes. For fitting of the survival function corresponding to $\psi _{T}(t)$ as shown in Fig. 2B, we consider a piecewise power-law, where the crossover timescale corresponds to the characteristic escape time of the bacterium from the trap. Although these choices of fit functions are physically intuitive, it is important to note that their specific form is not important for the modeling results. The predicted MSD and the long term motility characteristics such as the diffusion coefficient do not change for other choices of the fit functions as long as they match the observed waiting-time distributions reasonably well.

**Fig. 5:**
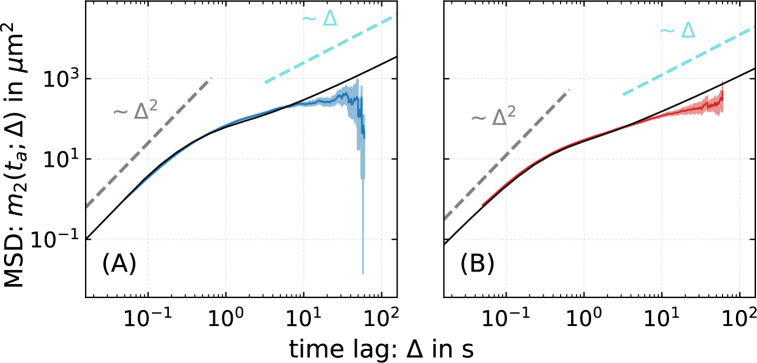
An active particle model explains the observed time dependence of the mean squared displacement of *P. putida* in agar, based on the experimentally observed motility pattern and the measured waiting-time distributions. Comparison of MSDs predicted by the model (black lines) with experimental data for *P. putida* wild-type cells in (**A**) $0.25\, \%$ agar (blue) and (**B**) $0.3\, \%$ agar (red). The shaded regions correspond to the error bars ($95 \, \%$ confidence interval) associated with the MSD obtained by bootstrapping^31^.

The parameters $v_0, D_\phi$, $\langle \cos \chi \rangle$ and those obtained from the fit of the waiting-time distributions can be readily derived from experimental data, enabling us to test the consistency of the model by comparing the experimentally observed MSD with the model at short time scales, and to predict long-time transport properties (for details of parameter estimation from the data, see Supplementary Note 1; parameter values are summarized in Supplementary Table S1).

In Fig. 5, we compare the experimentally measured MSD of *P. putida* wild-type cells in $0.25 \, \%$ and $0.3 \, \%$ agar and the predictions of the model, yielding quantitative agreement over more than two orders of magnitude in time. We note that the experimentally determined MSD curve for large lag times is increasingly unreliable due to the small number of long trajectories. In the long-time limit, the model predicts normal diffusion which is not resolved by the current experimental setup. Note that the diffusive regime at long times is not observed in our data due to experimental limitations (finite length of trajectories). With increasing time, it becomes more likely that typical bacterial swimming trajectories will leave the field of view accessible by a conventional microscopy setup. For this reason, long-time recordings selectively focus on trajectories of trapped or less motile cells that remain in the field of view, thus concealing the crossover to normal diffusion.

The model provides an analytical expression of the long-term diffusion coefficient for the bacteria undergoing intermittent run-motility

5 $$\begin{aligned} D&= \frac{v_0^{2}}{2D_{\phi }} \frac{\langle t_{R} \rangle }{\langle t_{R} \rangle +\langle t_{T} \rangle }\left[ 1 - \frac{1-\hat{\psi }_{R} \! \left( D_{\phi }\right) }{\langle t_R \rangle \!D_{\phi }} \frac{1-\langle \cos \chi \rangle }{1- \langle \cos \chi \rangle \hat{\psi }_R \! \left( D_\phi \right) }\right] \! , \end{aligned}$$

where $\langle t_R \rangle$ and $\langle t_T \rangle$ are the mean values of the corresponding waiting times (cf. caption of Fig. 2). We predict the long-term diffusion coefficients $D \approx 5.25 \, \upmu {\text {m}}^2 / \text {s}$ and $2.56 \, \upmu {\text {m}}^2 / \text {s}$ for wild-type *P. putida* cells in $0.25\, \%$ and $0.3 \, \%$ agar, respectively, as well as $8.55 \, \upmu {\text {m}}^2 / \text {s}$ for $\Delta$*motAB* and $6.81 \, \upmu {\text {m}}^2 / \text {s}$ for $\Delta$*motCD*, both in $0.25 \, \%$ agar.

## Discussion

Single cell tracking of bacterial motion in complex environments has been a rapidly growing topic over the past years. Examples include the swimming of bacteria, such as *E. coli* or *Vibrio fischeri*, in two-dimensional chambers of different heights^40,41^, in narrow channels^42,43^, microfluidic mazes^44^, and in suspensions of micro-spheres^45^. Swimming bacteria have also been combined with lyotropic liquid crystals to form a novel type of living liquid crystal^46,47^. For the soil bacterium *P. putida* considered in this work, swimming between planar interfaces^48^ and in microfluidic chambers with cylindrical obstacles has been previously investigated^49^. Besides well-defined microfluidic geometries, bacterial swimming has also been studied in disordered porous and gel-like substrates. In a porous matrix, formed by jammed packings of soft particles, *E. coli* moved in directed paths that were interrupted by transient mechanical trapping events in narrow spacings or cavities^7,8^, affecting also the chemotactic navigation strategy within the porous material^50^. Chemotaxis of the marine pathogen *Vibrio alginolyticus* was analyzed in agar hydrogels, showing that lateral flagella may prevent mechanical trapping and thereby improve the chemotactic performance^11^. Along with these experimental studies, also models were proposed to describe bacterial motility in heterogeneous disordered environments^10,34,51–56^.

Our present study of *P. putida* motility in agar hydrogels showed that cells display intermittent run motility. As shown previously, the average swimming speed is only moderately decreased^28^ in comparison to motion in bulk liquid. The duration of trap events, during which bacteria are effectively immobile, are, however, increased by a factor of 10 on average compared to swimming in bulk liquid and show a power-law distribution with a steep cutoff towards large times. Indications for similar power-law scalings have been observed for *E. coli* swimming in a porous material^7,8^. However, in contrast to the micron-sized openings between the surfaces of jammed particles that were used in these previous studies, cells propagate though the disordered meshwork of an agar hydrogel in our case. The run times in our data are 10 times shorter compared to bulk swimming and exhibit an exponential distribution, see Fig. 2. The mean trap time in agar, on the other hand, is similar to the mean run time of the bacteria in bulk fluid. This leads us to argue that bacteria typically escape from traps by actively initiating a turning maneuver. This is also in agreement with reports from *E. coli* showing that cells often tumble before leaving a surface^57^.

Our observations suggest that the swimming pattern in agar is affected by additional random trapping events in the polymer matrix. An analysis of the turn angle distribution for events with short and long dwell times supports this hypothesis (see Fig. 3). Together, these features of the swimming pattern result in a time evolution of the MSD that shows, after an initial ballistic regime, a (transient) sublinear scaling, see Fig. 1B. This is reminiscent of subdiffusive behavior typically observed for transport in disordered environments^58–60^. Examples include a wide range of different systems, such as passive particles diffusing in mucus or cytoskeletal networks^61,62^, water molecules at membranes^63^, active colloids in crowded environments^64^, or transport in a lipid bilayer^65^.

Previously, we demonstrated that colonies of the $\Delta$*motCD* mutant show a slower macroscopic spreading in agar as compared to $\Delta$*motAB* and wild-type colonies, due to the formation of sessile clusters, which reduce the number of motile cells^28^. On the level of the individual swimming trajectories, however, we now observed that both mutant strains display a similar time dependence of the MSD as the wild-type, despite changes in the swimming speed and in the frequencies at which the individual swimming modes occur. Specifically, we found that run times are longer on average for the slow swimming $\Delta$*motAB* mutant, resulting in a run length distribution that is identical to the distributions of the faster swimming $\Delta$*motCD* and wild-type cells. This provides additional evidence that the geometry of swimming trajectories in the gel is primarily imposed by the surrounding matrix and depends only to a lesser extent on details of the swimming pattern.

To account for the experimentally observed scalings in the MSD, we rely on a recently proposed active particle model that is based on renewal theory, in which the active agents intermittently switch between two states: a run state and immobile phases (events), during which active motion is absent but particles reorient their direction of motion^34^. We performed extensive data analysis to extract the distributions of the run and event duration as well as all other required parameters, such as the run speed, rotational diffusion coefficient and turn angles from data. When supplied with the experimentally derived parameters and distributions, our model closely matches the experimental findings. In particular, as a consequence of the power-law scaling in the waiting-time distribution $\psi _T(t)$, the model recovers the transition from ballistic to a sublinear crossover regime observed in our data. However, due to the cutoff of the power-law at large times, this regime remains transient. In the long time limit, normal diffusion is predicted; we explicitly estimate the long-time diffusion coefficient on the basis of the phenomenological active particle model.

Dynamical switching between motile and resting phases has been reported for a range of different systems, such as active colloidal particles in the presence of AC fields^66,67^, stick-and-slip dynamics of myxobacteria^68^, near-surface swimming of *E. coli*^69^, or navigation strategies of larger organisms^70^. Also on a conceptual level, similar dynamics has been considered for diffusive particles that switch between mobile and immobile states^71^ or for active Brownian particles alternating between stop and go phases^34,72–74^. Here, we have shown that bacterial swimmers in a gel matrix follow a similar intermittent run dynamics that can be described by a renewal process with power-law distributed dwell times. Given that many common bacterial habitats exhibit gel-like properties, we expect these findings to advance our quantitative understanding of how bacterial swimmers invade complex environments such as the human gut or the plant rhizosphere. How this movement strategy impacts chemotactic navigation in heterogeneous environments remains an open question to be addressed in future work.

## Methods

### Cell preparation

The strain *P. putida* KT2440 FliC_S267C_ was used in this study. The introduction of a surface-exposed cysteine residue in the flagellar subunit FliC enables fluorescent staining of the flagella without a negative effect on swimming motility. Therefore, we refer to this strain as the wild-type. Additionally the *P. putida* KT2440 FliC_S267C_ $\Delta$*motAB* and $\Delta$*motCD* mutant strains were used to study the swimming motility in agar. The $\Delta$*motAB* and $\Delta$*motCD* stator mutants were generated previously^28^. The strains were grown overnight in LB media as a shaking culture at $30 \,^{\circ } \text {C}$ in all experiments except for fluorescent staining experiments where tryptone broth (10 g/l tryptone [Applichem], 5 g/l NaCl) was used. Flagellar staining was performed following the protocol established in an earlier study^27^, using the fluorescent dye Alexa 488 C_5_ maleimide (Thermo Fisher Scientific). Additionally, the cell body was stained by adding $10 \, {\upmu }$l FM 4-64 (Thermo Fisher Scientific; $1 \, {\upmu }$g/${\upmu }$l in dimethyl sulfoxide) before the final washing step. Semisolid agar was prepared with 210 mM Na_2_HPO_4_, 220 mM KH_2_PO_4_, 50 mM NaCl, 1 mM MgSO_4_, $0.2\, \%$ glucose, $0.5\,\%$ casamino acids and $0.25 \, \%$ or $0.3 \,\%$ agar^75^. Agar was poured into FluoroDish cell culture dishes with glass bottoms. After 4 hours for solidification, $2 \, \upmu$l cells at an OD$_{600 \text {nm}}$ of one were injected into the agar. Fluorescence images were recorded after additional four hours. Phase contrast recordings were done at the next day when cells spread circularly from the injection point.

### Microscopy

Microscopy was performed in agar at the edge of the spreading culture using an inverted microscope (Olympus IX71) with a high-speed camera (Orca-Fusion BT digital CMOS camera, Hamamatsu) and the Hokawo software (Hamamatsu). Phase contrast recordings were taken 30 $\upmu$m above the bottom surface with a 20x objective (UPLFLN-PH, Olympus) for 60 seconds at a frame rate of 20 frames per second. Fluorescence images were made close to the bottom surface with a 60x objective (UPLFLN-PH, Olympus) for 10 seconds at a frame rate of 100 frames per second. To separate the cell body from the flagellar fluorescent signal, the W-View Gemini image splitting optics (Hamamatsu) was added to the microscopy setup.

## Supplementary Information

Supplementary Information 1.

Supplementary Information 2.

Supplementary Information 3.

Supplementary Information 4.

Supplementary Information 5.

Supplementary Information 6.

## Data Availability

The data that support the findings of this study are available from the corresponding author upon reasonable request.
