# Supplementary material for: Bacterial swimming in porous gels exhibits intermittent run motility with active turns and mechanical trapping
Source: Sci Rep. 2025 Jun 27;15:20320. doi: 10.1038/s41598-025-02741-1 (PMC12205082; doi:10.1038/s41598-025-02741-1)
Supplement: Supplementary file 6 — Supplementary Information 6. [file 41598_2025_2741_MOESM6_ESM.pdf]

SUPPLEMENTARY INFORMATION

Bacterial swimming in porous gels exhibits intermittent run motility with active turns and mechanical trapping

Agniva Datta, Sönke Beier, Veronika Pfeifer, Robert Großmann, and Carsten Beta

SUPPLEMENTARY NOTE 1: ANALYSIS OF TRAJECTORIES

In the following, we show how model parameters were obtained from experimental data and, in addition, discuss the criterion which was used to identify run episodes. We show the results for all the three strains of *P. putida* in 0.25 % agar (wild-type, $\Delta motAB$ and $\Delta motCD$).

Cell tracking. To perform segmentation and cell tracking, we used the method described in Ref. [1], which is based on Ref. [2].

Distinction of runs and events. To distinguish between the run phases and the immobile phases (events) in the recorded trajectories, at first we used the method proposed in Ref. [3]: a speed threshold was used to distinguish between run and event phases. We set this threshold to 7 µm/s (a little bit lower then the half of the slowest swimming wrapped mode in bulk [4]). We labeled all data points with a speed under 7 µm/s as events. Fig. S1A reveals that this criterion is not sufficient to detect the events. In many instances, one true event is broken down into several smaller events, which are interrupted by short runs. This is because, in between the trap events, the bacteria often swim back and forth. As a result, the measured speed of a bacterium may be faster than the threshold value for a short period of time.

We resolved this problem by setting a minimum distance (2 µm: comparable to the length of the bacterium) between two consecutive events in addition to the speed threshold; two consecutive events which are less than 2 µm apart from each other were fused, including all intermediate points of the trajectory. In Fig. S1B it can be seen that this detection works much more reliably on our data. Slightly different speed thresholds had no effect on the quality of the detection.

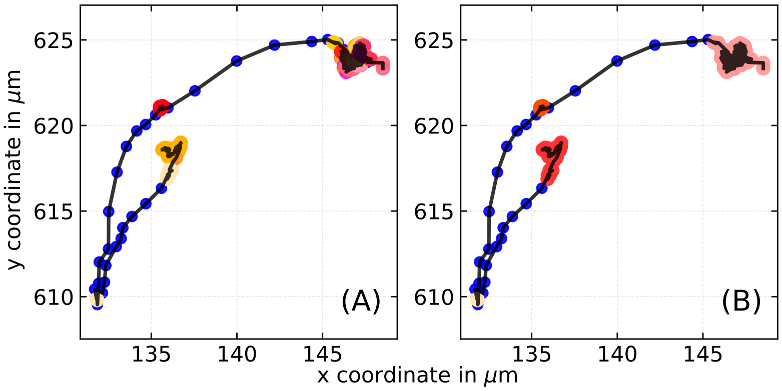

Figure S1. Trajectory comparison: Run phases are marked in blue. The events have different (randomly chosen) colors. (A) Only the velocity threshold of 7 µm/s is used. This leads to a detection of multiple events at a specific cluster of data points which clearly correspond to one event. (B) The minimum distance criterion of 2 µm along with the velocity threshold of 7 µm/s is used. This leads to a fusion of all the points in a specific cluster, corresponding to one event, thereby resulting in a better distinction of runs and events.

We plotted the distributions of the angular changes during runs (by considering the change in direction of motion between the beginning and end of the runs) and that of events (by considering the change in previous run direction compared to that of the next run direction after an event). Just by the velocity threshold and the minimum distance criterion, we can observe a significant difference in these angular change distributions for runs (peak in zero; Fig. S2A) and turn events (peak in 180°; Fig. 3 in the main text). However, for the runs, we clearly observe a smaller but significant peak in 180° as well, which clearly indicates that we are missing some short turn events (reversals) which contribute significantly to the overall motility pattern.

To have a more robust and precise run and event distinction, we use an algorithm based on unsupervised machine learning. At first, we smooth the trajectories over 3 data points (moving average). Afterwards, the following char-

acteristics are calculated for each point in a trajectory: change of direction of motion at a given data point for a short time interval, change of direction of motion over a longer time interval at that data point, and mean of the speeds incoming and leaving that data point. From a list of potential other observables, we obtained these three characteristics as the most relevant ones for the distinction of runs and events by principle component analysis. Then, we perform a k-means clustering on these three variables in order to distinguish the data points into two distinct categories: run phases and events (turns, stops, traps, reversals). In addition, we use the minimum distance criterion of 2 µm to fuse close by events to obtain the final labels for every data-point in the trajectories in terms of runs and events. It can be seen in Fig. S2B that it not only improves the angular change distribution plots for runs (the peak in 180° disappears, signifying no misdetections) but also does not require the velocity threshold parameter (7 µm/s) that we had to impose for the previous distinction algorithm. We use these labels for further analysis as described below.

We note that trajectories were not smoothed for the analysis of bacterial motility in 0.3 % agar as run times are significantly shorter and runs may be lost by smoothing.

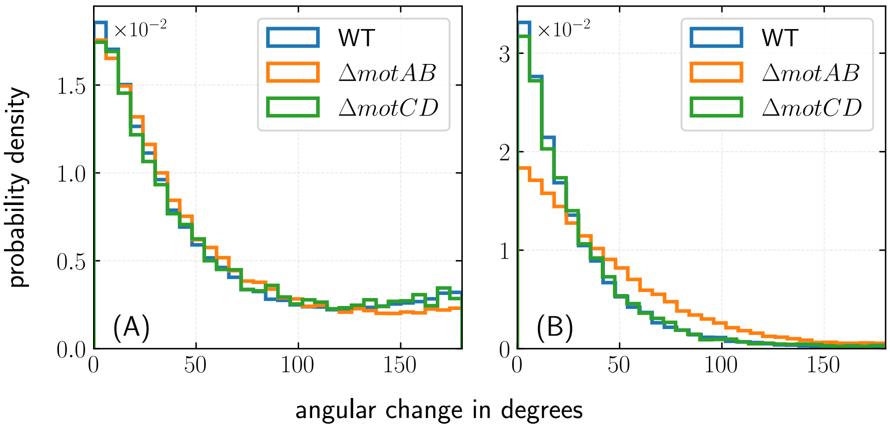

Figure S2. Comparison of angular changes during runs. (A) Distinction algorithm using the minimum distance criterion of 2 µm along with the velocity threshold of 7 µm/s. A small peak at 180° suggests that some short turn events were not detected (particularly reversals). (B) Distinction algorithm using k-means clustering leading to a better distinction where the peak at 180° is absent.

Run time and dwell time distributions. To obtain the run time and dwell time statistics in form of survival time probabilities as shown in Fig. 2 in the main text, we classify the recorded waiting-times into four categories: both, beginning and end of an interval are observed; only the end of a phase is observed; only the beginning of a phase is observed; neither the beginning nor the end of a phase are observed. Besides the first category, all intervals are censored. Then, we apply a non-parametric maximum likelihood approach proposed by Vardi in Ref. [5] to extract the survival time probabilities corresponding to the run time and dwell time distributions.

We fit the survival probability of the run time distribution using the survival function of a double-exponential distribution with a minimum time defined as follows:

$$\psi_R(t) = \begin{cases} 0 & t \le t_0, \\ p\kappa e^{-\kappa(t-t_0)} + (1-p)\lambda e^{-\lambda(t-t_0)} & t > t_0. \end{cases} \tag{S1}$$

We obtain the parameters p, κ, λ, t_0 from the fit of $\Psi_R(t) = \int_t^\infty \psi_R(x)\,dx$. Similarly, we fit the survival probability of the dwell time distribution using the the survival function of a piecewise power law with a minimum time defined as follows:

$$\psi_T(t) = \begin{cases} 0 & t < \tau_1, \\ At^{-\alpha} & t \in (\tau_1, \tau_2), \\ Bt^{-\beta} & t > \tau_2, \end{cases} \tag{S2}$$

where $A = \dfrac{(\alpha-1)(\beta-1)(\tau_1\tau_2)^\alpha}{(\beta-1)\tau_1\tau_2^\alpha + (\alpha-\beta)\tau_1^\alpha\tau_2}$ and $B = A{\tau_2}^{\beta-\alpha}$ are chosen such that $\psi_T(t)$ is normalized and continuous. The parameters of this distribution were obtained by fitting the corresponding survival functions $\Psi_T(t) =$

$\int_t^\infty \psi_T(x)\,dx$, shown in Fig. 2 of the main text and Fig. S3. Here, the parameters t_0 and τ_1 correspond to the minimum run time and minimum dwell time, respectively, which are similar to the temporal resolution of our microscopy setup and therefore serve as a regularization.

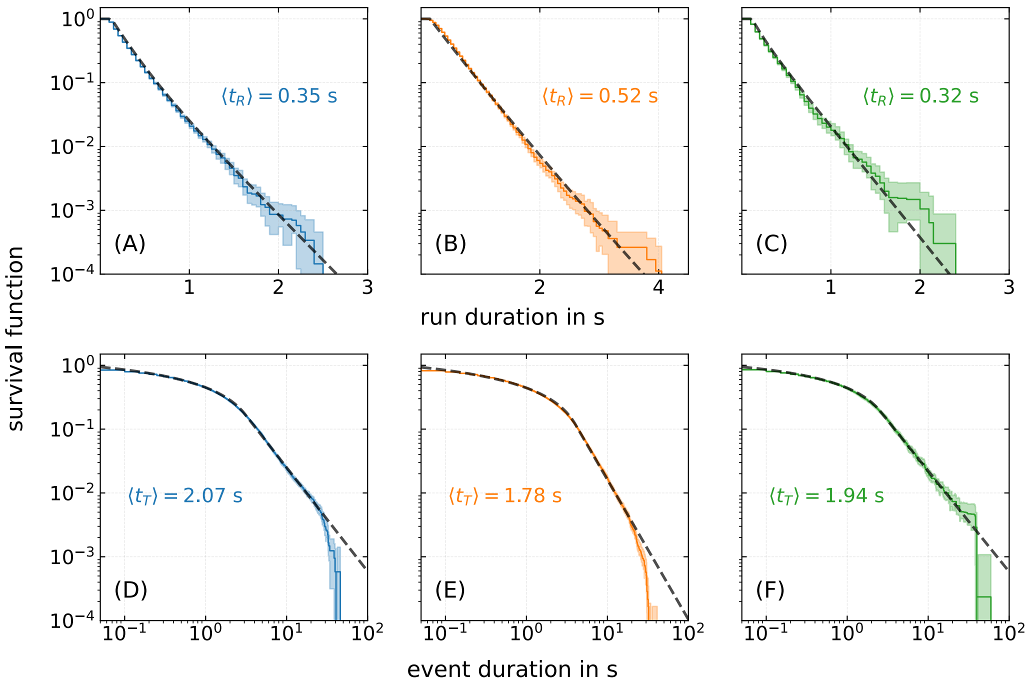

Figure S3. In the top panels (A-C), the survival functions corresponding to the run time distribution $\psi_R(t)$ for *P. putida* wild-type (WT), Δ*motAB*, Δ*motCD* along with their respective fits are displayed. In the bottom panels (D-F), the survival functions corresponding to the dwell time distribution $\psi_T(t)$ for wild-type, Δ*motAB*, Δ*motCD* along with their respective fits are shown. In all panels, the mean values of the probability distributions are given.

We further computed the mean run time $\langle t_R \rangle$ and the mean dwell time $\langle t_T \rangle$ as shown in Fig. S3 from the expressions of $\psi_R(t)$ and $\psi_T(t)$:

$$\langle t_R \rangle = \int_0^\infty t \cdot \psi_R(t)\,dt = t_0 + \frac{p}{\kappa} + \frac{1-p}{\lambda}, \tag{S3a}$$

$$\langle t_T \rangle = \int_0^\infty t \cdot \psi_T(t)\,dt = \frac{A\left(\tau_2^{2-\alpha} - \tau_1^{2-\alpha}\right)}{2-\alpha} - \frac{B\tau_2^{2-\beta}}{2-\beta}. \tag{S3b}$$

We obtained the mean run time and mean dwell time $(\langle t_R \rangle, \langle t_T \rangle)$ of $(0.35, 2.07)$ s for wild-type cells in 0.25 % agar, $(0.23, 3.63)$ s for wild-type in 0.3 % agar, as well as $(0.52, 1.78)$ s for Δ*motAB* and $(0.32, 1.94)$ s for Δ*motCD*, both in 0.25 % agar.

Rotational diffusion coefficient D_ϕ and tumble parameter $\Gamma = \langle \cos\chi \rangle$. Experimentally observed runs possess a finite persistence length. Angular fluctuations during run episodes are taken into account in the model via rotational Brownian motion, cf. Eq. (1) in the main text. This implies that the angular change of the direction of motion in between two consecutive frames follows a Gaussian distribution

$$P(\phi, \Delta t) = \frac{1}{\sqrt{4\pi D_\phi \Delta t}} e^{-\frac{\phi^2}{4D_\phi \Delta t}} \tag{S4}$$

with the variance $\langle \phi^2 \rangle = 2D_\phi \Delta t$. We consider all the run episodes of all trajectories and plot a histogram of the angular changes at each frame. A normal distribution is fitted to the histogram. From the variance of the fitted Gaussian curve, we obtain the rotational diffusion coefficient D_ϕ. This is illustrated in Fig. S4 for all strains.

Further, the tumble parameter $\Gamma = \langle \cos\chi \rangle$ is calculated as the mean cosine of the change of direction of motion between two consecutive runs—it is obtained from the distributions shown below (Fig. S5).

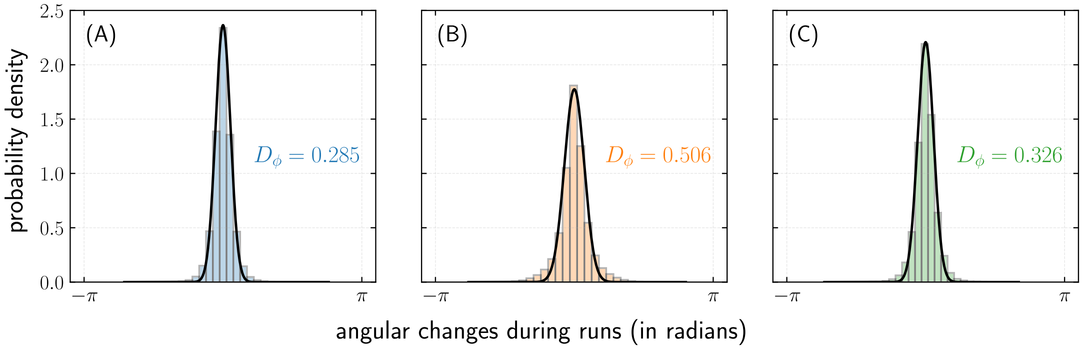

Figure S4. The rotational diffusion coefficient D_ϕ is obtained by fitting a normal distribution to the histogram of angular changes during runs for (A) *P. putida* wild-type cells (WT), (B) Δ*motAB* and (C) Δ*motCD*. The units of D_ϕ as shown are expressed as s^{-1}.

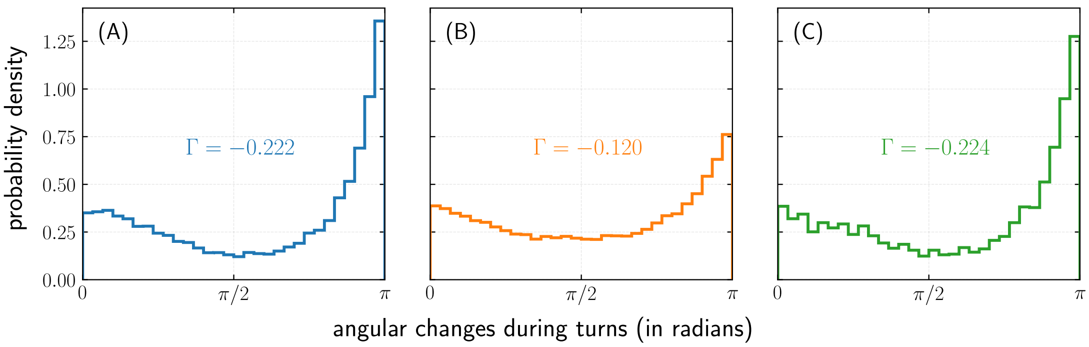

Figure S5. The tumble parameter $\Gamma = \langle \cos \chi \rangle$ is obtained by computing the mean obtained from the distributions of angular changes during turn events for (A) *P. putida* wild-type cells (WT), (B) Δ*motAB* and (C) Δ*motCD*.

Run speed v_0. The run speed parameter v_0 can be obtained in many different ways. However, since the MSD curves show a ballistic behaviour universally for small time lags reflecting self-propelled motion, we infer it by fitting the ballistic part of the MSD with a power law ($m_2 \sim \Delta^2$); note that the MSD scales with $v_0^2\Delta^2$ for small time lags Δ in our model [6]. We verify that the inferred values are consistent with the run speed distribution of bacteria as shown in Fig. S6.

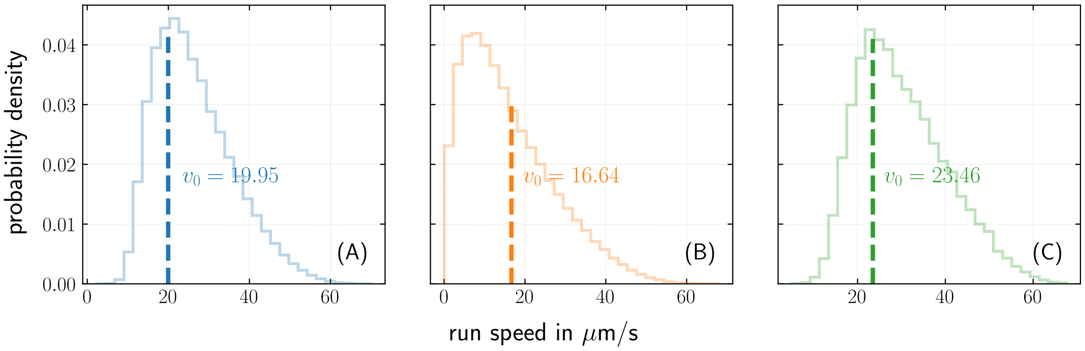

Figure S6. The run speed distribution of (A) *P. putida* wild-type (WT), (B) Δ*motAB* and (C) Δ*motCD*. The dotted lines correspond to speed values obtained by fitting the ballistic part of the MSDs (see Fig. 5 in the main text and Fig. S7). The units of v_0 as shown are expressed as µm/s.

SUPPLEMENTARY NOTE 2: MSD AND DIFFUSION COEFFICIENT

We finally plug in all inferred parameters (see Table S1) in the analytical expression of the MSD as stated in Eq. (4) in the main text. Subsequently, a dual inverse Laplace transform is performed numerically to predict the MSD in time domain [6, 7] and plot it as a function of the lag time Δ in comparison to the MSD curves obtained from experimental data (Fig. S7). The second temporal variable, which is the aging time t_a, is approximated to be one frame—it is a floating parameter that we cannot directly measure from the experiment. We get a very consistent match of our analytical expression of the MSD with the experimentally observed MSD for all strains at the experimentally recorded timescale. In the long-time limit, the model predicts normal diffusion as discussed in the main text.

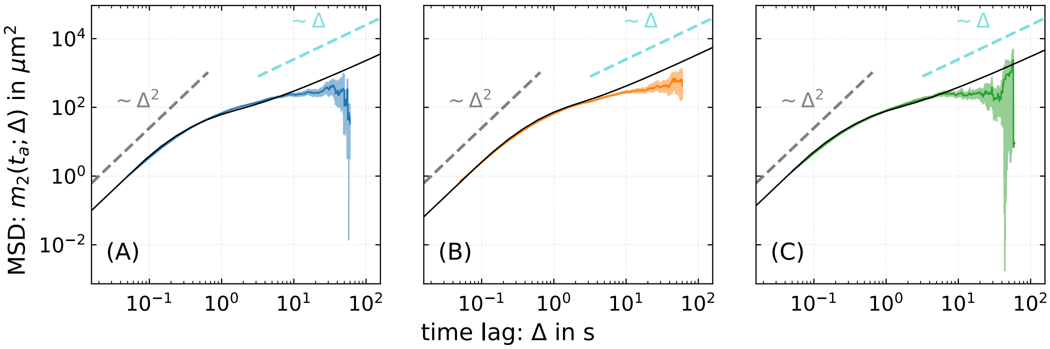

Figure S7. MSD predicted by the model for inferred parameter values, compared to the experimental MSD in color for (A) *P. putida* wild-type, (B) $\Delta motAB$ and (C) $\Delta motCD$. The patches correspond to the error bars (95 % confidence interval) obtained by bootstrapping [8].

From Eq. (5) in the main text, we determine the long-term diffusion coefficients by plugging in all the parameters we obtained from the data analysis as described in Supplementary Note 1 (cf. Table S1).

**strain**	v_0	D_ϕ	$\Gamma = \langle \cos\chi \rangle$	ψ_R [Eq. (S1)]				ψ_T [Eq. (S2)]			
				t_0	κ	λ	p	τ_1	τ_2	α	β
WT	19.95	0.29	−0.22	0.13	6.07	3.27	0.61	0.03	3.06	0.79	2.63
$\Delta motAB$	16.64	0.51	−0.12	0.15	3.07	2.31	0.62	0.03	4.01	0.83	3.19
$\Delta motCD$	23.46	0.33	−0.22	0.12	9.77	4.00	0.29	0.03	2.56	0.75	2.58

Table S1. The table summarizes all inferred parameter values, obtained from the analysis as described in Supplementary Note 1 for *P. putida* cells in 0.25 % agar. Parameters are expressed in the following units: v_0 in μm/s; D_ϕ, κ and λ in s^{-1}; t_0, τ_1 and τ_2 in s.

For the wild-type in 0.3 % agar, we inferred the following parameter values: $v_0 = 19.49\,\mu\text{m/s}$, $D_\phi = 1.33\,\text{s}^{-1}$, $\Gamma = -0.25$, $t_0 = 0.08\,\text{s}$, $\kappa = 8.04\,\text{s}^{-1}$, $\lambda = 3.37\,\text{s}^{-1}$, $p = 0.99$, $\tau_1 = 0.02\,\text{s}$, $\tau_2 = 3.95\,\text{s}$, $\alpha = 0.68$, $\beta = 2.70$. For the analysis of this dataset, we put the floating parameter t_a to be five frames.

SUPPLEMENTARY NOTE 3: TURN ANGLE DISTRIBUTIONS

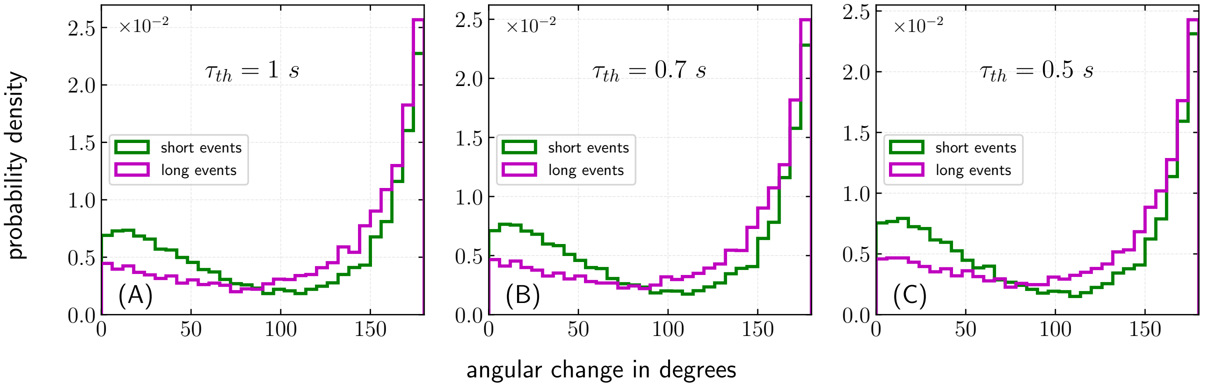

Figure S8. Turn angle distributions (cf. Fig. 3 in the main text) of short (shown in green) and long events (shown in purple) for *P. putida* wild-type in 0.25 % agar for threshold times (τ_{th}) of (A) $1\,s$, (B) $0.7\,s$ and (C) $0.5\,s$. Events with durations less than τ_{th} are considered to be short events and those with durations longer than τ_{th} correspond to long events. Qualitatively, the distributions do not depend on the chosen cutoff value.

SUPPLEMENTARY REFERENCES

[1] M. Theves, J. Taktikos, V. Zaburdaev, H. Stark, and C. Beta, A bacterial swimmer with two alternating speeds of propagation, Biophys. J. **105**, 1915 (2013).

[2] J. C. Crocker and D. G. Grier, Methods of digital video microscopy for colloidal studies, J. Colloid Interface Sci. **179**, 298 (1996).

[3] T. Bhattacharjee and S. S. Datta, Bacterial hopping and trapping in porous media, Nat. Commun. **10**, 2075 (2019).

[4] M. Hintsche, V. Waljor, R. Großmann, M. J. Kühn, K. M. Thormann, F. Peruani, and C. Beta, A polar bundle of flagella can drive bacterial swimming by pushing, pulling, or coiling around the cell body, Sci. Rep. **7**, 16771 (2017).

[5] Y. Vardi, Nonparametric estimation in renewal processes, Ann. Stat. **10**, 772 (1982).

[6] A. Datta, C. Beta, and R. Großmann, Random walks of intermittently self-propelled particles, Phys. Rev. Res. **6**, 043281 (2024).

[7] G. Doetsch, *Tabellen zur Laplace-Transformation und Anleitung zum Gebrauch*, Die Grundlehren der mathematischen Wissenschaften in Einzeldarstellungen mit besonderer Berücksichtigung der Anwendungsgebiete (Springer, 1947).

[8] B. Efron and R. J. Tibshirani, *An Introduction to the Bootstrap* (Chapman and Hall/CRC, New York, 1994).
